# Chromosome 1p and 6q Loss of Heterozygosity in Meningioma: A Comprehensive Analysis of the Two Chromatin Remodeling Complex Subunits ARID1A and ARID1B

**DOI:** 10.3390/cancers18091325

**Published:** 2026-04-22

**Authors:** Manuel Hinsberger, Julia Becker-Kettern, Wiebke M. Jürgens-Wemheuer, Katrin Bartelmei, Ralf Ketter, Joachim Oertel, Walter J. Schulz-Schaeffer

**Affiliations:** 1 Institute for Neuropathology, Medical Faculty, Saarland University, Building 90.3, 66421 Homburg, Germany; m.hinsberger@gmx.net (M.H.); julia.kettern@uks.eu (J.B.-K.); wiebke.wemheuer@uks.eu (W.M.J.-W.); katrin.bartelmei@uni-oldenburg.de (K.B.); 2 Department of Neurosurgery, Medical Faculty, Saarland University, Building 90.3, 66421 Homburg, Germany; ralf.ketter@uks.eu (R.K.); joachim.oertel@uks.eu (J.O.)

**Keywords:** ARID1A protein level, SWI/SNF complex, ELISA, LOH 1p, LOH 6q, meningioma grading

## Abstract

**Background/Objectives**: Loss of heterozygosity (LOH) in meningioma has been known for more than two decades. It has been shown that LOH on chromosome 1p36 is an independent marker of meningioma recurrence and progression. *ARID1A*, a tumor suppressor gene located on chromosome 1p36.11, is part of the chromatin-regulating *SWI/SNF* complex whose subunits are altered in 20% of cases across all tumor entities. **Methods**: Using our newly developed indirect enzyme-linked immunosorbent assay (ELISA), we investigated whether tumors with or without LOH 1p differ in ARID1A expression in 61 meningiomas. To study possible links between ARID1A and ARID1B, we tested for LOH 6q in association with LOH 1p using a PCR-based microsatellite approach. *ARID1B*, another member of the *SWI/SNF* complex, is located on 6q25.3. Additionally, we compared our ELISA results with immunohistochemistry data staining of ARID1A in tissue sections known to harbor LOH 1p. **Results**: Our results indicate that meningiomas harboring LOH 1p have significantly lower ARID1A levels compared to tumors without LOH 1p. In free nuclear protein fractions, reductions were up to 32% (CI: 6–58.7%). Interestingly, we found that ARID1A levels were significantly lower in tumors with recurrence and/or multiple localizations. In addition, our analysis of chromosome 6q uncovered a significantly strong correlation between LOH 1p and LOH 6q (*p* < 0.0001). **Conclusions**: These results highlight the importance of ARID1A in meningioma malignization and indicate for the first time functional evidence for LOH 1p.

## Simple Summary

It has been known for several years that a loss of heterozygosity (LOH) on chromosome 1p, especially LOH on chromosome 1p36, is an independent marker of meningioma recurrence and progression. We aim to identify a factor that may be responsible for this effect. The *ARID1A* (AT-rich interactive domain-containing protein 1A) gene is located on chromosome 1p36.11 and is part of the chromatin remodeling complex *SWI/SNF* (switch/sucrose non-fermentable complex). By demonstrating a possible link between a decreased ARID1A protein level and the LOH 1p, as well as recurrence and a higher tumor grading in meningioma, we provide for the first time functional evidence of the LOH 1p. This may be of prognostic and therapeutic interest.

## 1. Introduction

The current classification for meningioma highlights the ongoing progress in molecular grading of tumor entities. The WHO 2021 Classification of Tumors of the Central Nervous System introduced for the first time molecular markers into meningioma diagnostics, i.e., homozygous deletion of *CDKN2A* and/or *CDKN2B* deletion, as well as *TERT* promoter mutation [1,2]. Histopathology still plays a central role in the diagnosis of grade 1 and 2 meningioma, but the most malignant grade 3 can be diagnosed by either histopathology or the two above-described molecular features [1,2].

In addition to these diagnosis-relevant features, research in recent years has shown that, in addition to smaller mutations, gross chromosomal alterations like losses of heterozygosity (LOHs) play a particularly important role [1,3,4]. Based on this, Ketter et al. (2007) found LOH 22q and 1p to be the most important factors for meningioma recurrence in their genetic progression score [4]. In a recent study (*n* = 527) frequent chromosomal losses of heterozygosity include 22q (61%), 1p (36%), 6q (21%), 14q/18q (both 19%), and 18p (17%), while the most important factors for meningioma recurrence were: 1p-, 6q-, 10q-, 18q-, 19p- or *CDKN2A/B*-loss [5].

Not only the location but also the chronological order of losses seems to be highly conserved during the progress of meningioma formation starting from arachnoid cells [4,6]. Ketter et al. (2007) proposed an oncogenetic tree mixture model, where the primary loss of heterozygosity on chromosome 22 is followed by LOH 1p and one out of two possible pathways ends up with LOH on chromosome 6 [4]. Further research showed deletion of 1p36 to be an independent marker of meningioma recurrence and progression [7]. Recent investigations indicated that a specific region of 1p36 is strongly associated with malignant tumorigenesis in meningioma, which encodes the *ARID1A* (*AT-rich interactive domain-containing protein 1A*) gene, located on chromosome 1p36.11 [8,9].

*ARID1A* is part of the *SWI/SNF* (switch/sucrose non-fermentable) chromatin-remodeling complex, which is encoded by a total of 29 genes and contains up to 15 subunits when assembled [10]. Its role is to facilitate transcription regulators to access chromatin by moving and ejecting nucleosomes by combining various subunits with different functions [11]. In terms of classification, the mammalian SWI/SNF family can be further divided into three subtypes: BAF (BRG1-associated factor complex), PBAF (polybromo BRG1-associated factor complex) and ncBAF (non-canonical BAF complex) [10,11]. The *ARID1A* gene is alternatively spliced into three isoforms, the longest of which is considered the canonical form with 2285 amino acids (aa.) [12]. The ‘nucleocytoplasmatic’ protein ARID1A [13] (242 kDa) contains two important domains: highly conserved AT-rich interacting domain (ARID; aa. 1017 to 1108; DNA binding properties [14]) and a C-terminal region that has been recently described in PFAM as BAF250_C (PF12031; aa. 1976 to 2231; protein–protein interactions [10]) [15,16,17]. ARID1B has more than 60% sequence identity with ARID1A and can be considered a paralog of ARID1A [18]. Its gene *ARID1B* is located on chromosome 6q25.3 [8]. Functionally, BAF complexes consist either of ARID1A or of ARID1B, like SMARCA2 and SMARCA4 [18]. Since these two proteins are alternative, mutually exclusive subunits in the BAF complexes, further studies have shown that the presence of either subunit determines the function of the entire BAF complex [19]. Both ARID1A and ARID1B connect the core and the ATPase module [10].

Subunits of the *SWI/SNF* complex are responsible for about 20% of all mutations in various tumor entities and *ARID1A* has the highest frequency [20,21]. The majority of cancer-associated mutations in *ARID1A* are inactivating [22], so considering LOH is of great interest. Kadoch et al. (2013) found multiple *SWI/SNF* subunits affected in a single tumor [21], and Wang et al. (2020) were able to show synergistic effects for *ARID1A* and *ARID1B* double loss in endometrial carcinoma cells, MFE-296 [23]. To our knowledge, there are no detailed investigations analyzing whether loss of heterozygosity on chromosomes 1p and 6q affects ARID1A protein expression. In meningioma, *ARID1A* can be termed a tumor suppressor gene, as case reports [24,25] and large studies by Williams et al. (2020) (*n = 850*) [26] and Gill et al. (2021) (*n = 255*) indicate the importance of a correct ARID1A dose and function [27,28]. Investigations on *ARID1B* in meningiomas are rare; only Harmancy et al. (2017) found *ARID1B* deletions to be enriched in atypical *NF2* meningioma (30% versus 10%) in their analyses of primary atypical meningioma (*n = 208*) [29].

Since there are many indications that LOH 1p is relevant in meningioma, we decided to test our recently published enzyme-linked immunosorbent assay (ELISA) to quantify ARID1A in meningioma and to assess whether ARID1A impairment is a significant factor in tumorigenesis [30]. In comparison to immunohistochemistry, the ELISA ensures objective, precise and accurate measurement of ARID1A. To determine the LOH 1p status of our meningioma sample set, we used a PCR-based microsatellite analysis as previously published by our group [9], which we extended further to 6q, where *ARID1B* is located. As the localization of meningioma, e.g., skull-base, convexity, or spinal, may be caused by different genetic patterns [31], we also analyzed ARID1A expression with regard to location. Additionally, the presence of recurrent and multifocal meningioma was considered with regard to a possible impact.

## 2. Materials and Methods

### 2.1. Sample Details and Preparation

Sixty-one human meningioma samples (Supplementary Data) from neurosurgery acquired between 2018 and 2021 were diagnosed according to the WHO classification of the tumors of the central nervous system. For all WHO grade 2 and 3 meningioma and WHO grade 1 tumors harboring LOH 1p, *TERT* promoter mutation and *CDKN2A/B* loss analysis was performed [1]. The inclusion criteria of our retrospective study were the following: patient age > 18 years, informed consent of patients, confirmed diagnosis of meningioma by two independent neuropathologists, and sufficient tumor material (>100 mg of fresh frozen tumor tissue). In addition, the study should include a high proportion of tumors with LOH 1p alongside tumors without LOH 1p as a control group, in order to provide meaningful results, but not necessarily reflecting the expected distribution of the different grades. A minimum of 26 patients for each group was determined by power analysis (see Section 2.5). We also recorded data for patients with recurrent meningioma in our outpatient follow-up regimen, based on a personalized treatment plan.

**Figure 1:**
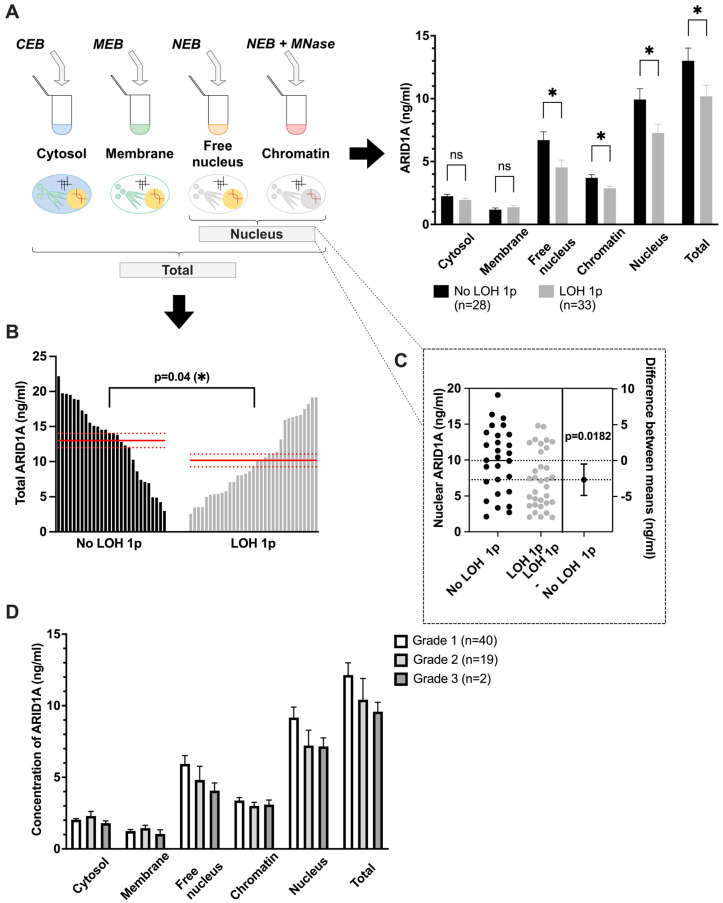
ARID1A expression is lower in meningioma with than without LOH 1p and lower in higher WHO grades. (**A**) Six different groups across several cellular compartments are analyzed to determine whether ARID1A is significantly reduced in LOH 1p meningioma (grey) compared to those with retained chromosome 1p (‘No LOH 1p’, black). Lysate preparation is shown on the left using ‘CEB’ = cytosol extraction buffer, ‘MEB’ = membrane extraction buffer, ‘NEB’ = nuclear extraction buffer’ and ‘MNase’ = micrococcal DNase, resulting in four fractions: cytosol, membrane, free nucleus, and chromatin. Nucleus = free nucleus + chromatin, and total = sum of all four fractions. Means and standard errors of the means (SEMs) are indicated. Exact values are shown in Table A2 . (**B**) Values of total ARID1A levels (sum of all subcellular fractions) are shown for 28 meningiomas with ‘No LOH 1p’ and 33 with chromosome ‘LOH 1p’. Means (black lines) as well as means ± SEMs (dotted lines) are indicated. Means (red solid lines) as well as SEMs (red dash lines) are indicated. (**C**) Scatter dot plot of ARID1A levels in group ‘nucleus’ is shown for meningiomas with LOH 1p (grey dots) and those with retained chromosome 1p (‘No LOH 1p’, black dots). On the right the difference between means (‘No LOH 1p’–‘LOH 1p’) ± SEMs is shown with the resulting *p*-value. (**D**) ARID1A levels are compared in all six cellular compartments regarding the three WHO grades 1 (white), 2 (light grey), and 3 (dark grey). Means ± SEMs are indicated.

Ethical approval was granted by the Ethics Committee of the Saarland Medical Council (no. 51/22). Fresh meningioma tissue was frozen in liquid nitrogen and stored at −80 °C. We processed samples prior to the analysis to obtain subcellular fractions using the ‘Subcellular Protein Fractionation Kit for Tissues’ (87790, Thermo Fisher Scientific, Waltham, MA, USA, RRID:SCR_008452) according to manufacturer’s instructions. This allows the analysis of subcellular fractions enriched in cytosolic, membrane-bound, free nuclear or chromatin-bound proteins. As previously described [30], ARID1A is predominantly observed in both free nucleus and chromatin, and to a minor extent in the cytosolic but not in the membrane fraction, which is in line with previous results by Guan et al. (2012) who reported a ‘nucleocytoplasmatic’ phenotype [13]. This supports the functionality of the fractionation kit in both tissue lysates and cell culture pellets. However, carry-over from one fraction to another cannot fully be excluded. The two subcellular compartments, free nuclear and chromatin-bound, were, as indicated for some analyses, combined into ‘nucleus’, and cytosol, membrane, free nucleus, and chromatin were summed up and termed ‘total’ for illustrative purposes (see Figure 1A). Localization of the tumors was noted, as by Maiuri et al. (2019) [32].

### 2.2. DNA Extraction

DNA was isolated from native tumor tissue, formalin-fixed paraffin-embedded tumor tissue and EDTA blood using the QIAamp DNA Micro Kit according to the manufacturer’s instructions (56403, Qiagen, Hilden, Germany, RRID:SCR_008539).

### 2.3. Microsatellite Analysis

For polymerase chain reaction (PCR)-based LOH analysis, primer pairs binding at various microsatellite loci were obtained from Eurofins Genomics (Ebersberg, Germany). Genomic positions for all primers binding on chromosomes 1p [9] and 6q are listed in Table A1.

Based on a protocol by Hartmann et al. (2005) routinely used in the neuropathology laboratory, we made minor modifications for our primers [33]. For each sample, 1 µL primer mix (forward and reverse primer, each 20 pmol/µL, Eurofins Genomic, Ebersberg, Germany) of the corresponding microsatellite was pipetted into 12.5 µL HotStarTaq Master Mix Kit (203445, Qiagen, Hilden, Germany) and 10.2 µL water, as well as 1.3 µL DNA (tumor and blood DNA in separate reactions). PCR cycling conditions were 94 °C for 15 min followed by 42 cycles at 94 °C for 30 s, 53 °C or 56 °C (see Table A1) for 40 s, and 72 °C for 40 s, followed by a final elongation step of 5 min at 72 °C in the Eppendorf Mastercycler nexus PCR Thermal Cycler (Eppendorf, Hamburg, Germany, RRID:SCR_023266).

PCR products were confirmed on a FlashGel^TM^ System using FlashGel^TM^ DNA Cassettes, 2.2% (57032, Lonza, Basel, Switzerland, RRID:SCR_00037). Subsequently, 1.5 µL PCR products were separated on a Spreadex^TM^ EL 800 Wide Mini (3446, AL-Labortechnik & Diagnostik GmbH, Zeillern—Amstetten, Austria) in 1× Tris-acetate-EDTA buffer (42548.01, TAE buffer (40×), Serva, Heidelberg, Germany, RRID:SCR_001063) at 120 V and 54 °C for 100 min in a horizontal gel electrophoresis system (Origins by Elchrom^TM^ Scientific, AL-Labortechnik & Diagnostik GmbH, Zeillern—Amstetten, Austria). Gels were stained with SYBR-Gold [0.8× TAE, 0.8× Destaining solution (3037.01, Serva, Heidelberg, Germany), 2× SYBR Gold, (S11494, Thermo Fisher Scientific, Waltham, MA, USA)] for 30 min. Images were acquired under UV light using the ‘EOS Utility’ program (Canon, Tokyo, Japan). If PCR products from tumor and blood had similar relative intensities of the two allelic bands, the allele was considered heterozygous. Loss of one band, as indicated by a lower signal intensity of one band in the tumor, was considered LOH. Stained gels were evaluated by two individual specialists in a double-blinded manner; if either or both expressed uncertainty, a third specialist was consulted. If a clear consensus could be reached, probes were classified as LOH or non-LOH. In any case of uncertainty, the sample was classified as non-evaluable (see Table A1).

### 2.4. Enzyme-Linked Immunosorbent Assay

Levels of ARID1A in meningioma tissue were determined by enzyme-linked immunosorbent assay (ELISA) as previously described [30]. Since the present study was designed simultaneously with the validation of our previously published enzyme-linked immunosorbent assay (ELISA) for ARID1A [30], sensitivity and specificity analyses had already been performed, including Western blot analyses for *ARID1A*-wildtype/-knockout and tumor lysates (Figure A3). However, it was not within the scope of this paper to fully re-validate the ELISA and Western blot results on a high sample number or cross-validate in another laboratory setting; therefore, interpretation of the results should be made carefully. In summary, qualitative controls included a standard curve consisting of 8 calibrators (inter-assay accuracy: 90.26%; inter-assay precision: 4.53%; and intra-assay precision: 4.05%) and six recurring tissue lysates as quality controls with low, medium, and high ARID1A expression on each plate (inter-assay precision: 10.61%).

Briefly, protein-binding plates were coated with tumor lysates, blanks, and standards of ARID1A fragment and incubated at 4 °C overnight. After blocking with casein, primary antibody directed against ARID1A (ab182560, Abcam, Cambridge, UK, RRID:AB_3096240) was added and incubated at room temperature for 2 h. HRP-conjugated detection antibody (K4003, Agilent Dako, Santa Clara, CA, USA, RRID:AB_2630375) was added. Absorbance at 490 nm was determined on the FLUOstar Omega Microplate Reader (BMG Labtech, Ortenberg, Germany, RRID:SCR_025024) after the addition of substrate and sulfuric acid and absorbances were analyzed with the MARS software V4.01 (BMG Labtech, Offenburg, Germany, RRID:SCR_021015). Day-to-day control samples and negative controls were included to control the variability of the assay.

### 2.5. Immunohistochemistry

Fresh samples were formalin-fixed, dehydrated and embedded in paraffin. One to three µm tissue sections were cut and applied to glass slides. After deparaffinization, antigen retrieval was performed in a steamer with Dako Target Retrieval Solution (pH 9, S2368, Agilent Dako, Santa Clara, CA, USA, RRID:SCR_013530). In the following stages, samples were incubated for 30 min with an anti-ARID1A antibody (diluted 1:1000, host species rabbit, ab182560, Abcam, Cambridge, UK, RRID:AB_3096240) in DAKO REAL antibody diluent (22022, Agilent Dako, Santa Clara, CA, USA, RRID:SCR_013530) according to the standard protocol for the EnVision+ System-HRP Rabbit/Mouse K5007 Kit (K5007, Agilent Dako, Santa Clara, CA, USA, RRID:AB_2888627), which was then applied to visualize the antibody reaction with 3, 3′-diaminobenzidine (DAB). Finally, images were acquired using the program ‘Leica Application Suite Version 3.8′ (Leica Microsystems GmbH, Wetzlar, Germany, RRID:SCR_016555).

### 2.6. Statistics

Data analysis was performed using GraphPad Prism (version 10.2.0 for Mac, GraphPad Software, San Diego, CA, USA, RRID:SCR_002798). The D’Agostino & Pearson test was used for normal distribution, with a *p*-value > 0.05 indicating normal distribution. Unpaired parametric *t*-tests were performed on Gaussian-distributed groups, while the Mann–Whitney test was used as a non-parametric test. Concordance between biomarkers (LOH of 1p and 6q) in tumor tissue and serum was assessed by Fisher’s exact test using contingency tables. When comparing values in more than two groups ANOVA (Analysis of Variance) was applied if those were Gaussian-distributed. If not, Kruskal–Wallis test was used. To assess the most important variables and adjust for confounders multiple linear regression was used. A *p*-value less than 0.05 was considered significant.

A priori power analysis was performed using an unpaired two-tailed *t*-test (α = 0.05, target power = 0.8, standardized effect size = 0.8), indicating a required sample size of at least 26 samples per group (52 in total). This corresponds to an achieved power of 0.807 using Prism. Post hoc analysis of 61 included patients revealed a strong power of 0.866.

## 3. Results

### 3.1. Study Cohort

The mean age of the patient population is 65.5 years (see Table 1). The age range is 29–92 years with a median of 66 years. Overall, 18% (*n = 11*), 56% (*n = 34*), and 26% (*n = 16*) are aged ≤ 54, 55–74, and ≥75 years, respectively (see Table 1 and Supplementary Data). Our study cohort includes 38 women (62%) and 23 men (38%). Among the women, 27 (71%), 10 (26%), and 1 (3%) have grade 1, 2, and 3 meningioma, respectively, compared to 13 (56%), 9 (39%), and 1 (5%) among the men. The overall female-to-male ratio is 1.65:1, varying considerably between low-grade meningioma (2.08:1) and high-grade tumors (WHO grade 2 and 3; 1.1:1). For further information on sociodemographic, disease-related or meningioma-specific aspects, please refer to Table 1 or see the Supplementary Data for additional information. Among the recurring meningiomas (in eleven, the first and second surgery dates are available), the average duration to recurrence was approximately 42 months and the median duration was approximately 38 months (minimum ~12.5 months, maximum ~73 months).

### 3.2. ARID1A Levels Are Lowered in Meningioma with LOH 1p and in High-Grade Meningioma

ARID1A concentrations were determined by an ELISA in different subcellular compartments, as described in Materials and Methods. The subcellular compartments due to fractionation are: cytosol, membrane, free nucleus, and chromatin. Comparison between the fractions reveals the highest concentrations in free nucleus, followed by chromatin, cytosol, and membrane in descending order (see Figure 1A). Free nucleus, chromatin, nucleus, and total show significantly lower levels (*p* < 0.05; *) compared to meningioma with intact chromosome 1p (n = 28; ‘No LOH 1p’, Figure 1A). Both cytosol and membrane fractions show no statistically significant differences.

Nuclear and total ARID1A levels are significantly reduced by 26.8% (CI: 4.7–48.8%), and 21.8% (CI: 1–42.5%), respectively, in meningioma harboring LOH 1p (compare Figure 1B,C and Table A2).

When comparing ARID1A levels across the different WHO grades independent of LOH 1p status (see Figure 1D), ARID1A levels decrease with higher WHO grades in the nucleus (median 9.2 ng/mL, 7.3 ng/mL, 6.8 ng/mL in WHO grade 1, 2, and 3, respectively) and total. However, this trend is not significant in any subcellular localization (*p*-value > 0.05; ANOVA for cytosol and membrane, Kruskal–Wallis test for others).

### 3.3. ARID1A Concentrations Are Decreased in Multifocal and Recurrent Meningioma

Between non-skull base meningioma and skull base meningioma, the cytosol fraction alone showed a statistically significant ARID1A decrease (*p* = 0.0323; *) (see Table 2). Comparison of spinal meningioma with non-skull base meningioma does not reveal any significant differences.

However, multifocal meningioma reveals a statistically significant reduction compared to non-skull base meningioma in nucleus and total ARID1A levels with 56.9% (CI: 10.4–82.5%) and 55.3% (CI: 17.7–83.1%), respectively (both *p* < 0.01; **). Interestingly, comparison of skull base meningioma with spinal meningioma and multifocal meningioma shows no statistically significant changes. Multifocal meningioma indicates a significant reduction in the cytosol, free nucleus, and chromatin fraction of 42.3% (CI: 3–82%), 68.7% (CI: 14.7–123%), and 35.7% (CI: 13.4–58.1%), respectively, compared to spinal ones. However, this trend is not visible in nucleus and total.

A more powerful analysis demonstrates that multifocal meningiomas (M) have 57.3% (CI: 10–90%) and 55.8% (CI: 11.9–80.6%) less ARID1A in nucleus or total compared to singular meningiomas, regardless of their localization (SI; sum of groups SB, N, and S). In addition, both findings are significant (*p* < 0.05) (see Table 2).

The 14 recurrent tumors are compared to the 47 primary tumors (see Table 3). Nucleus and total demonstrate statistically significant differences (*p* < 0.05; *) of 38.7% (CI: 1–62%), and 34.5% (CI: 1–58%), respectively, with less ARID1A in recurring meningioma (Table 3).

### 3.4. Different Immunohistochemical Pattern in ARID1A-Deficient Meningioma

**Figure 2:**
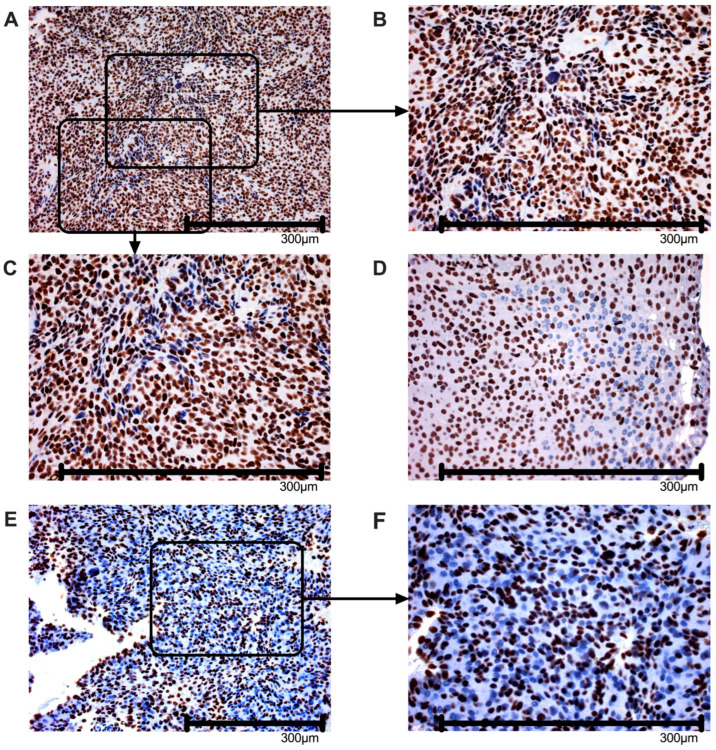
Immunohistochemistry analysis of ARID1A. (**A**–**D**) Minor mosaic-like loss of ARID1A expression. ARID1A-positive cells (stained brown) for antibody ab182560 (Abcam, Cambridge, UK) and ARID1A-negative cells (stained blue) are shown in patient 26 (**A**–**C**) and 1 (**D**). (**E**,**F**) Major mosaic-like loss of ARID1A expression. ARID1A-positive cells (stained brown) and ARID1A-negative cells (stained blue) are shown in patient sample 9. (**A**–**F**) The scale at the bottom indicates 300 µm. Using Fiji ImageJ [34] particle analysis was performed on the whole image using a size threshold of 20–infinity µm^2^ and circularity of 0.3–1.0, based on representative nuclear dimensions with the following results for ARID1A-negative cells: (**A**) = 21.7%; (**B**) = 48.2%; (**C**) = 45.1%; (**D**) = 19.2%; (**E**) = 60.2%; (**F**) = 56.8%.

To confirm the reduction in ARID1A levels in meningioma with LOH 1p, an immunohistochemical staining with the same antibody (ab182560, Abcam, Cambridge, UK), as used for the ELISA, was performed. Among all 33 tumors with LOH 1p, 13 (39%) meningiomas show a minor mosaic-like failure of ARID1A expression, as exemplified in Figure 2A–D. This contrasts with five (15%) meningiomas showing a major mosaic-like failure of ARID1A, where ARID1A-negative areas exceed 50% of total cells (see Figure 2E,F). In addition, 15 (46%) meningiomas harbor regular, homogenous ARID1A expression.

In our analysis none of the following parameters had a significant influence (*p* > 0.19) on ARID1A expression levels as determined by ELISA in a multivariate linear regression: gender, mitotic activity (via pHH3), and progesterone receptor, as well as features in the HE staining such as small cell changes, sheeting, or necrosis (Table A3). In addition, ANOVA reveals no differences in ARID1A when normal expression, minor or major mosaic-like loss is compared from IHC with ELISA results (*p*-value: 0.5592). Student’s *t*-tests of minor mosaic-like loss or major mosaic-like loss of ARID1A compared to tumors with retained expression show no significant reduction in ARID1A levels measured by ELISA. Due to a lack of correlation between IHC and ELISA, no IHC data from ‘No LOH 1p’ meningioma is added.

### 3.5. Partial Loss of Chromosome 6q Is Common in Meningioma

**Figure 3:**
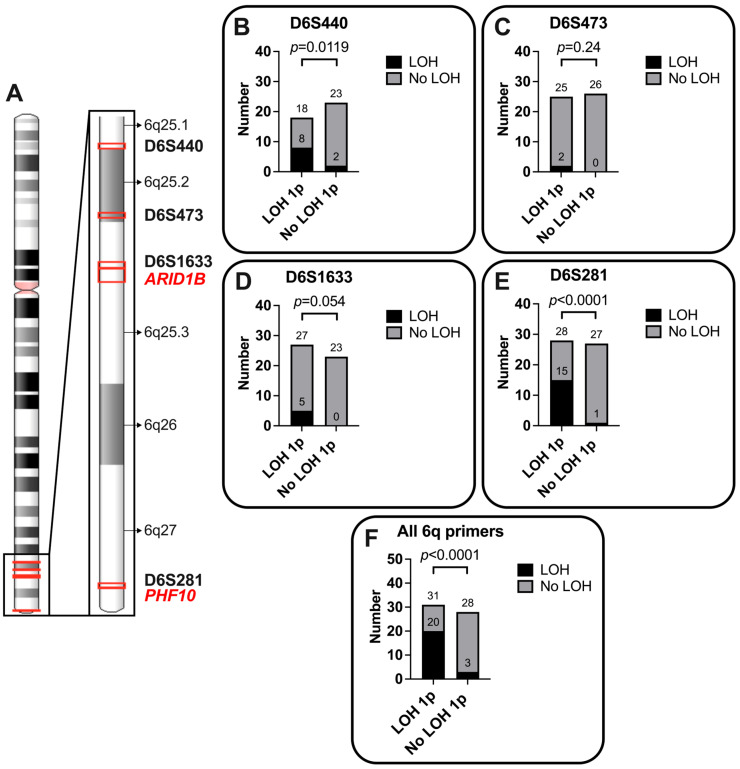
Loss of heterozygosity (LOH) analysis of the polymerase-chain reaction (PCR) probes for chromosome 6q. (**A**) Ideogram from the NCBI Genome Decoration Page shows the location of probes D6S440, D6S473, D6S1633, and D6S281, as well as the two subunits of the SWI/SNF complex *ARID1B* and *PHF10*, both highlighted in red color. Bands are named with an arrow on the right. (**B**–**E**) Analyses of probes D6S440, D6S473, D6S1633, and D6S281 are shown in independent boxes. The LOH of the corresponding probes is indicated in black (‘LOH’), while the retained probe in PCR is shown in grey (‘No LOH’). Resulting *p*-values are shown above the bars. (**F**) Summarizing loss of any of the four probes is termed ‘LOH’ (black) while no loss of any probe is termed ‘No LOH’ (grey).

To evaluate allelic losses on chromosome 6q, four microsatellites distributed across the long chromosomal arm were analyzed (Figure 3A). The informativeness rates for the primer pairs for chromosomal arm 1p varied between 82 and 90%, while the informativeness rate for chromosomal arm 6q was between 69 and 93%, giving in all cases a rather high degree of information (compare Table A1).

Loss of at least one of the four probes D6S281, D6S440, D6S473, or D6S1633 is found in 23 of the 59 (~39%) patient samples analyzed (Figure 3B–E). Complete loss of all four probes is only present in one patient. The loss of at least one of the four probes is statistically much more frequent in a tumor that also has an LOH on chromosome 1p compared to the group with two intact chromosome 1ps (Fisher’s exact test; *p* < 0.0001, Figure 3F). Among the tumors with LOH 1p, 20 of 31 (65%) have a loss of at least one of the four 6q probes, whereas this combination is seen in only four (11%) 6q probes of meningiomas without LOH 1p.

The microsatellite marker D6S281, which is located close to the end of the chromosome, is most frequently affected by LOH in 16 of 55 (~29%) evaluable patient samples (see Figure 3E, Fisher’s exact test; *p* < 0.0001). This marker is in close proximity to the SWI/SNF complex subunit *PHF10*. Chromosome 6q additionally contains the *ARID1A* paralog *ARID1B*.

## 4. Discussion

In this work, we analyzed ARID1A expression in 61 meningioma tissues using our newly developed indirect enzyme-linked immunosorbent assay (ELISA). Since we have demonstrated objective, precise, and accurate measurements on a small and defined subset of tumor samples as well as cell culture pellets and we have met the good-laboratory-practice guidelines (see Section 2) [30], our current focus lies on the transfer and applicability to human tumor tissue.

Holleczek et al. (2019) presented data from meningiomas of 992 patients from the federal state of Saarland, where our patient cohort of 61 samples also underwent surgery [35]. Epidemiological data suggest that our cohort appears to have a more aggressive course in comparison, as first, the recurrence in our cohort is 23% (compared to 6.1% by Holleczek et al. (2019)), second, the overall female-to-male ratio is 1.65:1 (2.53:1), and third, the proportion of multifocal meningioma is higher with 13% (*n* = 8) [35]. We see comparable values in age, WHO grade distribution [35] and localization [32]. Nevertheless, the high frequency of LOH 1p, with 54% compared to 36% by Driver et al. (2022) [5], reflects a shift towards malignancy in our cohort selection, as LOH 1p is an independent marker of meningioma recurrence and progression [7]. However, we primarily aimed to exceed the sample sizes for both LOH 1p- and No-LOH 1p-meningioma according to our power analysis and not necessarily only to match other epidemiological data.

**Figure A1:**
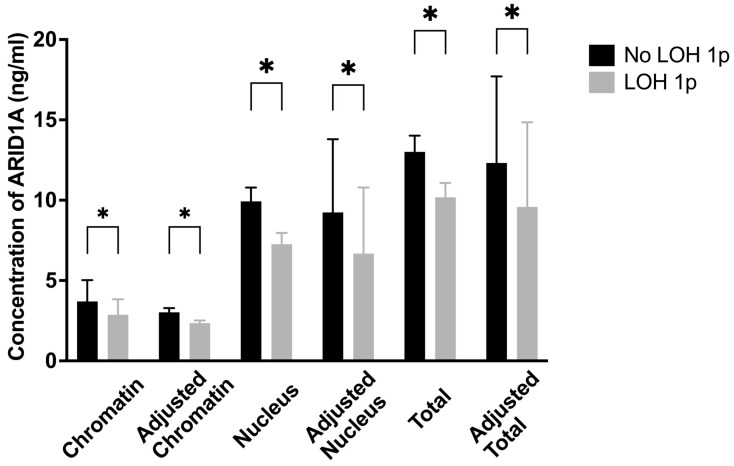
ARID1A expression in meningioma with and without LOH of chromosome 1p in an adjusted model. Six different groups across several cellular compartments are analyzed to determine whether ARID1A is significantly reduced in ‘LOH 1p’ meningioma (grey, *n *= 33) compared to those with retained chromosome 1p (‘No LOH 1p’, *n *= 28, black). This model for calculation includes correction for chromatin background (‘Adjusted Chromatin’, ‘Adjusted Nucleus’, ‘Adjusted Total’) . Means and standard errors of the mean (SEMs) are indicated. The asterisk (*) indicates *p* < 0.05. Exact values are shown in Table A4.

The comparison of 33 tumors with LOH 1p—thus also an allelic loss of the *ARID1A* gene located at 1p36.11 [8]—and 28 tumors without such a chromosomal loss shows that meningiomas with LOH 1p seem to contain significantly less ARID1A (compare Figure 1A–C and Table A2). As ARID1A is a ‘nucleocytoplasmatic’ partially DNA-bound protein [11,13], we suggest focusing on the free nucleus and chromatin fractions, because not all groups show significant changes (compare Table A2). Previous results strongly support our assumption; however, extensive validation of subcellular protein fractions in a large cohort remains to be done. Summing up to the two groups ‘nucleus’ (free nucleus + chromatin) and ‘total’ (cytosol + membrane + free nucleus + chromatin) should generally be considered the most interesting (Figure 1B,C) [30]. Therefore, significant decreases in the four groups, free nucleus, chromatin, nucleus, and total, do outweigh insignificant differences in cytosol and membrane fractions since the latter harbor generally lower ARID1A levels (compare Figure 1A). When considering the method for an adjusted model in chromatin fractions by mathematically reducing the threshold as described in [30], no changes occur (see Figure A1 and Table A4). Our data support the finding that *ARID1A* should be considered a driver gene [24,25,26,27,28], as the expression of ARID1A seems to be significantly reduced when LOH 1p is present, a state which is intimately linked with recurrence frequency and high grade [7].

**Figure A2:**
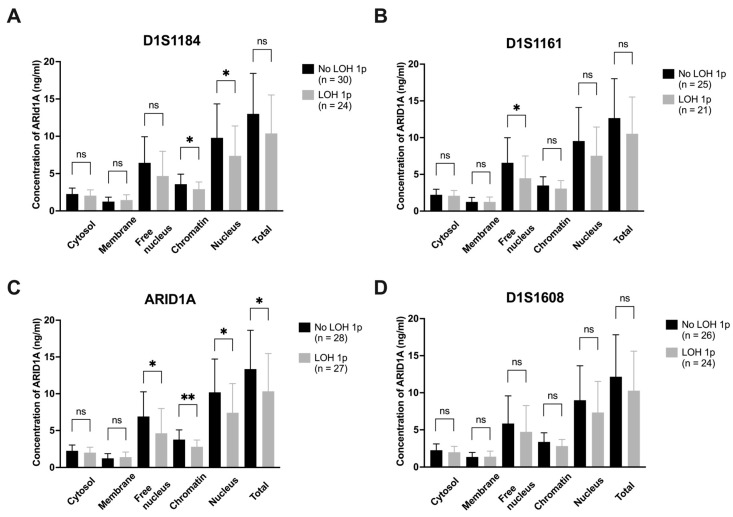
ARID1A expression in four microsatellites with and without LOH. (**A**–**D**) For each of the four microsatellites six different subcellular localizations are analyzed to determine whether ARID1A is significantly reduced in those meningioma harboring ‘LOH 1p’ (grey) compared to those with ‘No 1p LOH’ (black). Microsatellites D1S1184, D1S1161, ARID1A, D1S1608 are listed from centromere to telomere. For detailed localization see Table A1. Significance levels: * indicates a *p*-value between 0.01 < *p* < 0.05; ** indicates a *p*-value between 0.001 < *p* < 0.01; ns indicates a *p*-value > 0.05.

Since Ketter et al. (2007) found LOH 1p to be highly relevant [4] in recurrent meningioma our study is the first to link this chromosomal loss to a specific protein, thereby supporting already existing data on its gene *ARID1A* [7,9]. Loss of heterozygosity is a common way of inactivation in tumor suppressor genes [36]; therefore, we argue that haploinsufficiency appears to be a major underlying cause of lower ARID1A expression, as has also been shown in pancreatic cancer [37]. *ARID1A* mutation frequencies range from 5.4% [26] to 17.3% [27] in meningioma, but their influence on ARID1A expression has not been investigated. In addition, it is possible that hypermethylation of the *ARID1A* promoter via H3K27Me3 [38] influences ARID1A expression. Both mechanisms could add information about outlier values of meningioma without LOH 1p that have low nuclear ARID1A levels or tumors with LOH 1p having still high ARID1A expression (see Figure 1C), which seems to be a great opportunity for future investigations, as this manuscript does not provide this possible explanation for outliers. However, the 26.8% (CI: 4.7–48.8%) decrease in ARID1A nuclear protein level meningioma with LOH 1p (compare Table A2) indicates a functional relevance in our series of 61 meningiomas and underlines the haploinsufficiency (compare Figure 1A–C, Table A2). Especially when considering the loss of the *ARID1A* microsatellite as one part out of four investigated chromosome 1p microsatellites, we are able to support the above-described statements: loss of the *ARID1A*-microsatellite [9] seems to be tightly linked to a reduction in ARID1A expression as measured by our ELISA (see Figure A2C). In addition, our data suggest that neither LOH of microsatellite D1S1608 (located on 1p36.32) nor LOH of D1S1161 (1p35.2) leads to significant changes in ARID1A expression levels in any fraction (*p* > 0.05), a fact that is quite interesting and raises the possibility of *ARID1A* being mainly responsible in cases of LOH 1p (See Figure A2A, B, D).

Furthermore, the analyses of multifocal, recurrent meningioma indicate the reduction in ARID1A to be of great importance in patients’ outcomes (see Table 2 and Table 3). Our ELISA reveals that multifocal tumors have 55.8% (CI: 11.9–80.6%) less ARID1A in total compared to singular ones, which is not only statistically significant (*p* < 0.05) but should also be of functional relevance (see Table 2). In our series, ‘multifocal meningioma’ includes 63% recurrences (see Supplementary Data), which is different compared to the literature, where ‘multiple meningioma’ primarily addresses meningioma with multifocal locations [39]. In the data provided, these tumors express very low ARID1A levels and therefore seem to be of great interest in understanding the role of *ARID1A* in meningioma. Since the total group of recurrent meningioma harbors 34.5% (CI: 1–58%) less ARID1A compared to non-recurrent meningioma (*p* < 0.05), the ARID1A decrease should be of great importance to the patients’ outcomes, strengthening recent research about *ARID1A* mutations [24,25,26,27,28] and LOH 1p [3,4,5,7,40,41]. We argue that this effect of both multifocal and recurrent meningioma is mostly confounded by LOH 1p. However, when testing the influence of LOH 1p on ARID1A expression while adjusting for gender and WHO grade (both not significant in univariate analysis), as well as recurrence and localization (both significant in univariate analysis) in a multivariate analysis, we detect no significant trend (*p* > 0.05) in any of the parameters (compare Table A5).

**Figure A3:**
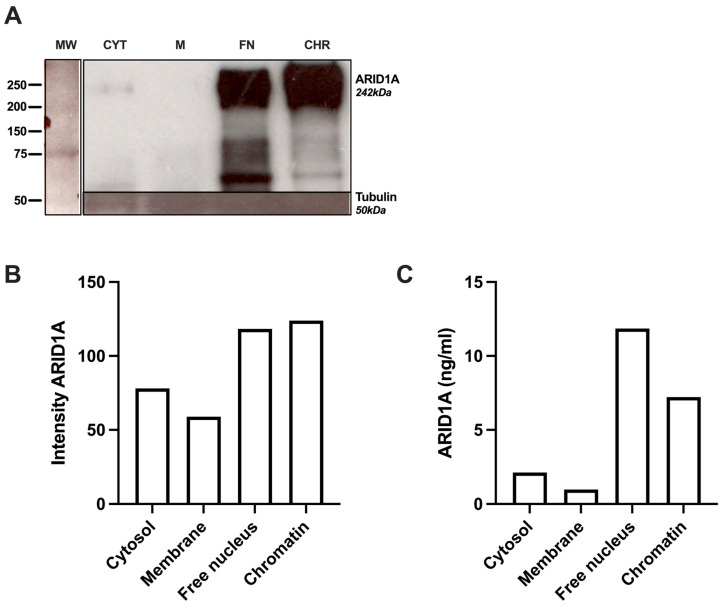
ARID1A expression in tumor lysate using Western blot. (**A**) The lysate of the tumor tissue (Case no. 37) is fractionated into cytosol (CYT), membrane (M), free nuclear (FN), and chromatin (CHR) fractions. Western blot analysis was performed according to our previous study [30]. Equal amounts of protein (30 µg) from tissue homogenates were separated by SDS-PAGE (4569033, Bio-Rad Laboratories, Hercules, CA, USA) and transferred to 0.2 µm nitrocellulose membranes (1620112, Bio-Rad Laboratories, USA) for 60 min at 100 V. Membranes were blocked overnight at 4 °C and incubated with primary antibodies against ARID1A (Ab182560, 1:2000, Abcam, UK) or α-tubulin (Ab7291, 1:2200, Abcam) as loading control, followed by HRP-conjugated secondary antibodies: K4003 (Dako, Glostrup, Denmark, diluted 1:500 in TBS-T) for ARID1A or anti-mouse antibody (K400111, Dako, Denmark, diluted 1:500 in TBS-T) for alpha-Tubulin, respectively. Signals were detected using ECL (RPN2235, VWR, Darmstadt, Germany) and visualized on hyperfilms (28-9068-37, VWR, Germany). Molecular weight markers were included in each run. Comparison with a molecular weight marker (MW, left lane) suggests the size of the intense band to be approximately 242 kDa, which matches full-length ARID1A (black arrow) or alpha-tubulin (50 kDa, blue arrow). An unspecific reaction at 16 kDa was observed, suggestive of MNase, as discussed in [30]. (**B**) Intensity measurement was performed using ImageJ, version 1.54p [34]. (**C**) Corresponding ELISA results of ARID1A levels measured in the lysates used in (**A**). The original Western blots are shown in Supplementary Figure S1.

Immunohistochemical staining (IHC) of ARID1A reveals minor as well as major mosaic-like ARID1A loss in 54.5% of meningioma cells with LOH 1p. While these patterns may represent the heterogeneity of ARID1A expression [6], neither is significantly associated with reduced ARID1A levels in ELISA (see Section 3.4). In line with other studies, we did not detect a total loss of ARID1A expression in any meningioma [24,25]. However, a cross-validation in a second independent cohort would further strengthen our results and possibly explain discrepancies in IHC and ELISA. Detailed analysis of our series shows that neither meningioma of WHO grade 2 or 3 nor recurrent or multifocal localized meningioma is significantly more frequent in tumors with or without mosaic-like ARID1A expression losses in IHC (Fisher’s exact test; *p* > 0.05). Therefore, we argue that ELISA should be considered as the method of choice to detect ARID1A levels in meningioma, as the limitations of a semiquantitative analysis in IHC outweigh the benefits of this method [30,42,43]. This is highly supported by our previous study confirming precise and accurate quantification of our ELISA using several Western blot assays [30], for example, in a representative meningioma lysate (see Figure A3).

*ARID1A* is a member of the SWI/SNF complex and Kadoch et al. (2013) were able to show that multiple subunits can be affected in a single tumor, a combination that can affect up to 50% of cases with SWI/SNF subunit alteration in a tumor entity [21]. In our analysis, we hypothesized that LOH 1p, which is often followed by LOH 6q [4], may lead to the loss of an allele of another subunit of the SWI/SNF complex, namely *ARID1B*. When considering the loss of any of the four chromosome 6q microsatellites (D6S281, D6S440, D6S473, and D6S1633), Fisher’s exact test shows that this loss is highly correlated with LOH 1p (*p* < 0.0001, Figure 3F). The single loss of polymerase chain reaction (PCR) probes D6S440 or D6S281 is shown to be significantly enriched in meningioma with LOH 1p (see Figure 3B,E). The latter is located very close to the *PHF10* gene—a tumor suppressor gene and another subunit of the SWI/SNF complex (compare Figure 3A) [44]. Since microsatellites within the *ARID1B* gene failed in our PCR, we used D6S1633 in the closest proximity to all markers to *ARID1B* for our analysis. Assuming LOH 6q reflects *ARID1B* impairment, interestingly, this probe very closely fails to meet significance with a *p*-value of 0.054 when analyzing the accumulation of LOH D6S1633 with LOH 1p (Fisher’s exact test; Figure 3E). Nevertheless, a bigger cohort might make this observation significant. Since there are strong indications of the malignant potential in tumorigenesis related to the double knockout of *ARID1A* and *ARID1B*, we propose to include multiple SWI/SNF members in mutational analyses in the future to better evaluate chromatin remodeling dysfunction in meningioma [19,20,21,23]. As Pérez-Magán et al. (2010) found most of the differentially expressed genes in their series were located at chromosomes 1p, 6q, and 14q and were under-expressed in recurrences (*n = 104*) [41]; the link of LOH, as well as mutation of *ARID1A* and/or *ARID1B* and additional members of the SWI/SNF complex (i.e., *PHF10* on 6q, *DPF3* on 14q), has the potential to expand the current understanding of meningioma development [45].

In order to consider potential influencing factors, we would like to address a few specific aspects. Since the data set presented was derived from real-world neurosurgical care practice over a period of more than three years, we considered its robustness and high statistical power of 0.866 to be major advantages. At the same time, its retrospective, single-center design limits generalizability [46]. Sociodemographic items, national peculiarities, selection bias and inter-observer reliability could have an influence on the study results. To limit these and other methodological biases, STROBE guidelines were followed closely, including careful definition of study variables, transparent reporting of inclusion and exclusion criteria, standardized data collection procedures, and comprehensive documentation of statistical methods to ensure reproducibility and minimize reporting bias [47]. As mentioned earlier, the influence of potential mutations or promoter methylation of the *ARID1A* gene is an additional factor that, alongside LOH 1p, could have a functional impact on ARID1A expression. We are aware that the current study does not address all questions; however, this was not the objective of a proof-of-principle study. Given these considerations, meningioma cell culture studies are desirable to confirm the impact of LOH 1p on protein expression. In addition, either native tumor tissue or cell culture assays using exome sequencing, quantitative PCR, methylation profiling and/or proteomics could help distinguish pathway-specific aspects.

Since ARID1A is involved in other signaling pathways such as AKT, DNA repair, TERT, HDAC, PD-L1, and cell cycle [48], it would be interesting to see whether there are interactions with impaired ARID1A signaling in meningioma and how they affect tumor growth, especially in high-grade tumors. As both *TERT* promotor mutation and homozygous deletion of *CDKN2A/B* have been included in the meningioma classification [1], and members of the SWI/SNF complex are subject to high mutation frequencies in some meningioma subtypes, such as *SMARCA4* (WHO grade 2 intraventricular meningioma [40]), *SMARCE1* (clear cell subtype [1]), *SMARCB1* (*NF2*-mutant meningioma [45]), *BAP1* (rhabdoid subtype [1]), and *PBRM1* (papillary subtype [1]), we argue that LOH 1p including the *ARID1A* gene and/or *ARID1A* inactivating mutation [22] with potentially decreased ARID1A levels should be the focus of further research and may be involved in meningioma grading. Consequently, we emphasize the necessity of prospective evaluation of LOH 1p meningioma and clinical outcomes or new treatment strategies such as *PARP*-inhibition [49] or *ATR*-inhibition [50], highlighting just a few promising ideas for future projects.

## 5. Conclusions

Loss of heterozygosity (LOH) 1p and especially loss of 1p36 is an independent marker of meningioma progression and recurrence. Since *ARID1A* (AT-rich interactive domain-containing protein 1A) is located on 1p36.11, its protein ARID1A, part of the mSWI/SNF complex, could be affected. Therefore, our aim was to show possible links of gene loss and consequently protein loss by using our newly developed indirect ELISA for ARID1A and several chromosome 1p probes. We observed that decreased ARID1A ELISA signals are associated with LOH 1p, recurrence and higher tumor grading in meningioma. Additionally, there is a significant clustering of both LOH 1p and LOH 6q, indicating possible interactions between ARID1A and ARIB1B, located on 6q25.3.

Since we provide for the first time a functional connection between LOH 1p and decreases in ARID1A protein levels measured by ELISA, research in the future may focus on prognostic and therapeutic implications. ARID1A is involved in many cell signaling pathways; therefore, patients with LOH 1p could be treated with personalized drugs.

## Figures and Tables

**Table 1:** Distribution of age, sex, recurrence, and WHO grade in 61 meningioma. Mean and standard deviation (SD) are shown for age in years (y). Values for sex, recurrence, WHO grade, and localization, as for Maiuri et al. (2019) [32], show absolute values as well as percentages according to the column in brackets. ‘M’ = meningioma, not specified; ‘ANGM’ = angiomatous meningioma; ‘MM’ = meningothelial meningioma’; ‘PM’ = psammomatous meningioma; ‘SM’ = secretory meningioma; ‘FM’ = fibrous meningioma; ‘AM’ = atypical meningioma; ‘CM’ = chordoid meningioma; ‘CCM’ = clear-cell meningioma; ‘ANM’ = anaplastic meningioma; ‘SB’ = skull-base meningioma.

		LOH 1p (*n = 33*)	No LOH 1p (*n = 28*)	Total (*n = 61*)
Age	Mean ± SD	62.2 ± 13.4 y	69.3 ± 13.7 y	65.5 ± 13.9 y
Sex	Female	20 (61%)	18 (64%)	38 (62%)
	Male	13 (39%)	10 (36%)	23 (38%)
Histological Subtype	M	16 (49%)	13 (46.5%)	29 (48%)
	ANGM	1 (3%)	2 (7%)	3 (5%)
	FM	0	1 (3.5%)	1 (1.5%)
	MM	1 (3%)	3 (11%)	4 (7%)
	PM	1 (3%)	1 (3.5%)	2 (3%)
	SM	0	1 (3.5%)	1 (1.5%)
	AM	10 (30%)	7 (25%)	17 (28%)
	CM	1 (3%)	0	1 (1.5%)
	CCM	1 (3%)	0	1 (1.5%)
	ANM	2 (6%)	0	2 (3%)
WHO Grading	Grade 1	19 (58%)	21 (75%)	40 (66%)
	Grade 2	12 (36%)	7 (25%)	19 (31%)
	Grade 3	2 (6%)	0	2 (3%)
Recurrence	Recurrent	11 (33%)	3 (11%)	14 (23%)
	Non-recurrent	22 (67%)	25 (89%)	47 (77%)
Localization	Skull-Base (SB)	5 (15%)	10 (36%)	15 (25%)
	Non-SB	21 64%)	13 (46%)	34 (56%)
	Spinal	0	4 (14%)	4 (6%)
	Multifocal	7 (21%)	1 (4%)	8 (13%)

**Table 2:** Analysis of ARID1A concentrations in different localizations. Nominal values of each localization are shown in brackets to enhance perception. The group of tumors with multifocal meningioma (M) has 57.3% less ARID1A expression with a simple significance of 0.01 ≤ *p* < 0.05 compared to the group of meningioma with singular foci (SI) in the nucleus (M vs. SI). Localizations M vs. S show strong reductions in the nucleus and total fractions of 65.3% and 62%, respectively, with both fractions falling far short of the significance level of *p* < 0.05, with *p* = 0.1535 and *p* = 0.2141.

Groups (n)	Cytosol	Membrane	Free Nucleus	Chromatin	Nucleus	Total
SB (15) vs. N (34)	−21.6% (*)	14.0%	8.6%	2.5% ^1^	−5.7% ^1^	−3.4%
S (4) vs. N (34)	2.5%	14.5%	54.7%	25.0%	24.5%^1^	9.2%
M (8) vs. N (34)	−43.7% (***)	16.7%	−51.6% (*)	−19.7% ^2^	−56.9% (**) ^1^	−55.3% (**) ^1^
S (4) vs. SB (15)	24.3%	−25.0%	42.4%	25.9% ^1^	15.5%	13.1%
M (8) vs. SB (15)	−28.2%	2.4%	−55.4% ^2^	−10.5% ^1^	−54.3% ^1;2^	−52.3% ^1;2^
M (8) vs. S (4)	−42.3% (*)	36.5%	−68.7% (*)	−35.7% (**)	−65.3% ^1^	−62.0% ^1^
M (8) vs. SI (53)	−40.0% (**)	78.5% ^1^	−71.6% (*) ^1^	−10.5% ^1;2^	−57.3% (*) ^1^	−55.8% (**) ^1^

^1^ Non-Gaussian-distributed groups, Mann–Whitney U test needed by using medians to calculate relative differences. ^2^ Failing to meet the significance level of *p* < 0.05, with *p*-values of *p* = 0.0601, *p* = 0.0755, and *p* = 0.0755, respectively, for free nucleus, nucleus, and total indicate potential relevance. SB = skull base, N = non-skull base, S = spinal, M = multifocal, SI = singular focus (all singular meningioma foci of the groups SB, N and S). Significance levels were: * indicates a *p*-value between 0.01 < *p* < 0.05; ** indicates a *p*-value between 0.001 < *p* < 0.01; *** indicates a *p*-value between 0.0001 < *p* < 0.001.

**Table 3:** Analysis of ARID1A levels in primary and recurrent meningioma. The comparison of ARID1A concentrations in six cell compartments’ dependence on the recurrence state is shown. Groups were tested for Gaussian distribution: if Gaussian-distributed, Student’s *t*-tests were applied and means were calculated. Accordingly, for non-Gaussian-distributed groups (indicated by ‘^1^’), Mann–Whitney U tests were applied and medians were calculated. Column 4 (‘Difference ± SEM’) contains the absolute difference with indication of the standard error of the mean (SEM) if applied. For non-Gaussian-distributed groups, medians are compared. The relative difference is calculated as a percentage from the absolute difference in the dependence on the mean (Gaussian-distributed groups) and median (non-Gaussian-distributed groups). Significance level *p* < 0.05 (*). Significant values are given in bold.

Fraction	No Recurrence (ng/mL), n = 49		Recurrence (ng/mL), n = 12		Difference ± SEM (ng/mL)	Relative Difference (%)	*p*-Value
	Mean	Median	Mean	Median			
Cytosol	2.14	N/A	1.89	N/A	−0.25 ± 0.25	−11.6%	0.3331
Membrane	1.30	N/A	1.16	N/A	−0.14 ± 0.28	−10.6%	0.6292
Free nucleus ^1^	N/A	6.36	N/A	2.45	−3.91	−61.4%	0.0460 (*)
Chromatin ^1^	N/A	3.24	N/A	2.6	−0.64	−19.7%	0.0107 (*)
Nucleus ^1^	N/A	9.09	N/A	5.58	−3.51	−38.7%	0.0401 (*)
Total ^1^	N/A	12.30	N/A	8.05	−4.25	−34.5%	0.0455 (*)

## Data Availability

Data were generated by the authors and included in the article. The added Microsoft Excel file contains all data used in this study; see the attached ‘Supplementary Data’.

## Supplementary Materials

The following supporting information can be downloaded at https://www.mdpi.com/article/10.3390/cancers18091325/s1: Supplementary Data (Excel file): Patient cohort and additional analyses summarized in an Excel file. The following variables are presented in the file. Number: Patient numbers from 1 to 61; Age: Patients age in years; Sex: M=male, F=female; Meningioma subtype: M' = meningioma, not specified, 'ANGM' = angiomatous meningioma, ' MM' = meningothelial meningioma', ' PM' = psammomatous meningioma, ' SM' = secretory meningioma, ' FM' = fibrous meningioma, ' AM' = atypical meningioma, ' CM' = chordoid meningioma, ' CCM' = clear-cell meningioma, ' ANM' = anaplastic meningioma; Brain infiltration: B' = brain infiltration, '-' = no brain infiltration; WHO grade_2016: WHO grade according to the WHO Classification of Tumors of the Central Nervous System (CNS), published in 2016; WHO grade_2021: WHO grade according to the WHO Classification of Tumors of the Central Nervous System (CNS), published in 2021; 'R' = Recurrent Meningioma, '-' = No recurrenent meningioma; Localization: Localization of the tumor according to radiologic classification Localization_2 According to Maiuri et al. (2019) sphenoid wing, olfactory and petroclival meningiomas are assigned to the skull base (SB). Tumors of the falx, frontal convexity, temporal convexity, occipital pole, and parietal pole are assigned to the convexity meningioma group (N; non-skull base). Additionally, there are spinal (S) and multiple meningiomas (M). The group of singular tumors (SI) is composed of: SB + N + S; *TERT*: *TERT* promotor mutation: 'C228T' = mutation, '-' = no mutation, 'NA' = data not available; *CDKN2A/B*: *CDKN2A/B* status: 'loss' = deletion of *CDKN2A/B*, '-' = no deletion, , 'NA' = data not available; LOH 22q: LOH'= loss of heterozygosity (LOH) of chromosome 22q, '-' = no LOH, 'NA' = data not available; LOH 14q: 'LOH'= loss of heterozygosity (LOH) of chromosome 14q, '-' = no LOH, 'NA' = data not available; LOH 1p: 'LOH'= loss of heterozygosity (LOH) of chromosome 1p, '-' = no LOH; LOH 1p_2: LOH of chromosome 1p in x out of y cases, where evaluation is possible: 'x/y'; D1S1608: 'LOH'= loss of heterozygosity (LOH) of D1S1608, '-' = no LOH, 'NA' = data not available, 'NE' = PCR not evaluable due to (a) Heterozygosity (b) PCR failure, i.e. blurred bands or incorrect PCR product length; *ARID1A*-LOH: 'LOH'= loss of heterozygosity (LOH) of *ARID1A*, '-' = no LOH, 'NA' = data not available, 'NE' = PCR not evaluable due to (a) Heterozygosity (b) PCR failure, i.e. blurred bands or incorrect PCR product length; D1S1161: 'LOH'= loss of heterozygosity (LOH) of D1S1161, '-' = no LOH, 'NA' = data not available, 'NE' = PCR not evaluable due to (a) Heterozygosity (b) PCR failure, i.e. blurred bands or incorrect PCR product length; D1S1184: 'LOH'= loss of heterozygosity (LOH) of D1S1184, '-' = no LOH, 'NA' = data not available, 'NE' = PCR not evaluable due to (a) Heterozygosity (b) PCR failure, i.e. blurred bands or incorrect PCR product length; LOH 6q: LOH of chromosome 6q in x out of y cases, where evaluation is possible: 'x/y'; D6S473: 'LOH'= loss of heterozygosity (LOH) of D6S473, '-' = no LOH, 'NA' = data not available, 'NE' = PCR not evaluable due to (a) Heterozygosity (b) PCR failure, i.e. blurred bands or incorrect PCR product length; D6S440: 'LOH'= loss of heterozygosity (LOH) of D6S440, '-' = no LOH, 'NA' = data not available, 'NE' = PCR not evaluable due to (a) Heterozygosity, (b) PCR failure, i.e. blurred bands or incorrect PCR product length; D6S281: 'LOH'= loss of heterozygosity (LOH) of D6S281, '-' = no LOH, 'NA' = data not available, 'NE' = PCR not evaluable due to (a) Heterozygosity, (b) PCR failure, i.e. blurred bands or incorrect PCR product length; D6S1633: 'LOH'= loss of heterozygosity (LOH) of D6S1633, '-' = no LOH, 'NA' = data not available, 'NE' = PCR not evaluable due to (a) Heterozygosity, (b) PCR failure, i.e. blurred bands or incorrect PCR product length; ARID1A_cytosol: ARID1A values of cytosol measured by ELISA, 'NE' = ELISA well not evaluable due to measurement below the quantitative range; ARID1A_membrane: ARID1A values of membrane measured by ELISA, 'NE' = ELISA well not evaluable due to measurement below the quantitative range; ARID1A_free nucleus: ARID1A values of free nucleus measured by ELISA, 'NE' = ELISA well not evaluable due to measurement below the quantitative range; ARID1A_chromatin: ARID1A values of chromatin measured by ELISA, 'NE' = ELISA well not evaluable due to measurement below the quantitative range; ARID1A_chromatin_corrected: ARID1A values of chromatin after correction for background in an advanced model measured by ELISA, 'NE' = ELISA well not evaluable due to measurement below the quantitative range; ARID1A_nucleus: ARID1A values of nucleus (free nucleus + chromatin) measured by ELISA, 'NE' = ELISA well not evaluable due to measurement below the quantitative range; ARID1A_nucleus_corrected: ARID1A values of nucleus (free nucleus + chromatin) after correction for background in an advanced model measured by ELISA, 'NE' = ELISA well not evaluable due to measurement below the quantitative range; ARID1A_total ARID1A values of total (cytosol + membrane + free nucleus + chromatin) measured by ELISA, 'NE' = ELISA well not evaluable due to measurement below the quantitative range ARID1A_total_corrected; ARID1A values of total (cytosol + membrane + free nucleus + chromatin) after correction for background in an advanced model measured by ELISA, 'NE' = ELISA well not evaluable due to measurement below the quantitative range; IHC: ARID1A loss of immunohistochemistry signal: 'A' = minor mosaic-like ARID1A loss, 'B' = minor mosaic-like ARID1A loss, '-' = no loss of ARID1A signal, 'NA' = data not available; Mitotic activity (pHH3): Mitotic activity measured by pHH3, numbers represents the count of cells positive for pHH3 per 400 HPF indicating mitotic activity; Ki-67: Ki-67 count in percent of cells per 400 HPF; PR: Progesterone receptor positivity via immune reactivity score: (IRS; 0–12) = IS (staining intensity; 0–3) * PP (percentage points; 0–4); SCC: Histological feature of small cell change (SCC): '+' = yes, '-' = no; Sheeting: Histological feature of sheeting: '+' = yes, '-' = no; Necrosis: Histological feature of necrosis: '+' = yes, '-' = no. Figure S1: Original Western blots for Figure A3. (A) The original Western blot of part A in Figure A3 incubated with an antibody against ARID1A and anti-alpha Tubulin for the adjustment of the amount of loaded proteins as explained in Figure A3. (B) Original Western blot of part A in Figure A3 incubated with an antibody against ARID1A for illustration purposes. ARID1A (black arrow), Tubulin (blue arrow) and MNase (red arrow) indicate the respective bands. The box below contains intensity measurements using ImageJ V1.54p [34] including an intensity ratio analysis.

## Abbreviations

The following abbreviations are used in this manuscript:

aa	Amino acids
ANOVA	Analysis of Variance
ARID1A	AT-rich interactive domain-containing protein 1A
BAF	BRG1-associated factor complex
DAB	Diaminobenzidine
ELISA	Enzyme-linked immunosorbent assay
FFPE	Formalin-fixed paraffin-embedded
IHC	Immunohistochemistry
LOH	Loss of heterozygosity
LOH 1p	Loss of heterozygosity on chromosome 1p
M	M multifocal meningioma
N	Non-skull base meningioma
ncBAF	Non-canonical BAF complex
No LOH 1p	No loss of heterozygosity on chromosome 1p
PBAF	Polybromo BRG1-associated factor complex
PCR	Polymerase chain reaction
S	Spinal meningioma
SB	Skull-base meningioma
SD	Standard deviation
SEM	Standard error of the mean
SI	Singular meningioma
SWI/SNF	Switch/sucrose non-fermentable complex

## Appendix A

**Table A1:** Microsatellites and probes for chromosome 1p and 6q. The detailed information for localization, DNA sequence, length, and annealing temperature is given for the microsatellites used for the polymerase chain reaction (PCR)-based loss of heterozygosity (LOH) analysis of chromosomes 1p (upper part) and 6q (lower part). All products used were produced by Eurofins Genomics (Ebersberg, Germany). To address reliability, we added informative rates for LOH 1p (eligible for 61 patients) and LOH 6q (eligible for 59 patients) by showing fractions of either LOH, No LOH, or not evaluable (NE) results, including nominal values in brackets.

Microsatellite	D1S1608	D1S1161	D1S1184	*ARID1A*
Binding region	1p36.32	1p35.2	1p31.3	1p.36.11
DNA sequence	5′GATGGCTTTTGGGGACTATT3′	5′AGAATGGTGTGAACCCAGG3′	5′AGCCAAGATCATGCCACTG3′	5′AACCCACTGTCTCTTTGGGAAGGC3′
	5′CACTGAGCCAAGTGACACAG3′	5′AAGGATTATACAGCAGCTGTT3′	5′CCTCCTGGCAAAATATCCAT3′	5′TGAGCCAAGATTGGA3′
Length	278 bp	299 bp	258 bp	222 bp
Annealing	53 °C	53 °C	53 °C	56 °C
Informativeness rate % (n)	LOH: 39.3% (24)	LOH: 34.4% (21)	LOH: 39.3% (24)	LOH: 44.3% (27)
	No LOH: 42.6% (26)	No LOH: 41% (25)	No LOH: 49.2% (30)	No LOH: 45.9% (28)
	Not evaluable (NE): 18% (11)	NE: 24.6% (15)	NE: 11.5% (7)	NE: 9.8% (6)
Microsatellite	D6S281	D6S440	D6S473	D6S1633
Binding region	6q27	6q25.1	6q25.2	6q25.3
DNA sequence	5′CTGGTAGTGTCAGGCATGGC3′	5′GCTAAGGATAACTTTCTGCTAGAC3′	5′AGAACTTGGTATTTCCTGCC3′	5′TCATGGAGCTTATAGCCTGG3′
	5′CCTATGTTTCAGGCAAAGGC3′	5′TTCTTCATTTTACGGATGG3′	5′GCCACCTTGAGGAGTTTT 3'	5′TTCCTTGATGTTATCAGGACAC3′
Length	209 bp	271 bp	184 bp	127 bp
Annealing	56 °C	56 °C	56 °C	56 °C
Informativeness rate % (n)	LOH: 27.1% (16)	LOH: 16.9% (10)	LOH: 3.4% (2)	LOH: 8.5% (5)
	No LOH: 66.1% (39)	No LOH: 52.5% (31)	No LOH: 83.1% (49)	No LOH: 76.3% (45)
	NE: 6.8% (4)	NE: 30.5% (18)	NE: 13.6% (8)	NE: 15.3% (9)

**Table A2:** Comparison of ARID1A concentrations in tumors with and without loss of heterozygosity (LOH) of chromosome 1p in six different cell compartments. The ARID1A concentrations are shown, with the mean or median values in columns 2 and 3. Groups were tested for Gaussian distribution: if Gaussian-distributed, Student’s *t*-tests were applied and means were calculated. Accordingly, for non-Gaussian-distributed groups (indicated by ‘^1^‘), Mann–Whitney U tests were applied and medians were calculated. Column 4 (‘Difference ± SEM’) contains the absolute difference with indication of the standard error of the mean (SEM) if applied. For non-Gaussian-distributed groups, medians are compared. The relative difference is calculated as a percentage from the absolute difference in the dependence on the mean (Gaussian-distributed groups) and median (non-Gaussian-distributed groups). Significance levels are given in bold; (*) indicates a *p*-value < 0.05.

Fraction	No LOH 1p (ng/mL)		LOH 1p (ng/mL)		Difference ± SEM (ng/mL)	Relative Difference (%)	*p*-Value
	Mean	Median	Mean	Median			
Cytosol	2.24	N/A	1.95	N/A	−0.29 ± 0.20	−13.0%	0.1518
Membrane ^1^	N/A	1.02	N/A	1.13	0.11	10.6%	0.5999
Free nucleus	6.7	N/A	4.53	N/A	−2.17 ± 0.88	−32.3%	**0.0172 (*)**
Chromatin ^1^	N/A	3.37	N/A	2.81	−0.56	−16.5%	**0.0104 (*)**
Nucleus	9.93	N/A	7.27	N/A	−2.66 ± 1.09	−26.8%	**0.0182 (*)**
Total	13.01	N/A	10.18	N/A	−2.83 ± 1.35	−21.8%	**0.04 (*)**

**Table A3:** Multivariate analysis of parameters in immunohistology influencing ARID1A concentrations in ELISA. Multiple linear regression model to assess significant parameters. In the two groups ‘nucleus’ and ‘total’ we analyzed whether one of the following parameters had significant (*p* < 0.05) influence of ARID1A measured by ELISA in each tumor: sex (M: Male; since women tend to have less often aggressive meningioma, male sex is seen as a risk factor, see Section 4), ARID1A loss with ab182560 (Abcam, UK) in immunohistochemistry (F: focal loss; M: mosaic-like pattern, see Section 3.4), mitotic activity (via pHH3; counted as X mitoses per 100 HPF), progesterone receptor (PR, indicated by immunoreactive score (IRS): counting of the stained cells (staining intensity 0–3) multiplied by the proportion of positive cells (percentage points 0–4).), as well as features in the HE staining such as small cell change, sheeting or necrosis, where the presence of (+) indicates a risk factor, see [1,2]. Estimates, standard errors, 95% confidence intervals (CIs) and t-values ‘|t|’ as well as *p*-values (significance level *p* < 0.05) are shown for each variable and for each of the six subcellular groups (cytosol, membrane, free nucleus, chromatin, nucleus, and total).

Fraction	Variable	Estimate	Standard Error	95% CI (Asymptotic)	|t|	*p*-Value
Nucleus	Sex [M]	−0.021	1.75	−3.67 to 3.63	0.0121	0.9905
	IHC [F]	1.10	2.02	−3.11 to 5.32	0.547	0.5908
	IHC [M]	3.58	2.84	−2.35 to 9.501	1.26	0.2221
	Mitotic activity	−0.045	0.151	−0.36 to 0.2694	0.300	0.7673
	PR	−0.191	0.28	−0.775 to 0.392	0.683	0.5022
	SCC [+]	−3.25	2.45	−8.36 to 1.86	1.33	0.1992
	Sheeting [+]	2.33	2.1	−2.05 to 6.71	1.11	0.2802
	Necrosis [+]	0.699	2.84	−5.22 to 6.62	0.246	0.8079
Total	Sex [M]	0.241	2.3	−4.55 to 5.03	0.105	0.9174
	IHC [F]	1.12	2.65	−4.41 to 6.66	0.424	0.6764
	IHC [M]	4.07	3.73	−3.71 to 11.85	1.091	0.2882
	Mitotic activity	−0.0568	0.198	−0.47 to 0.357	0.287	0.7774
	PR	−0.164	0.367	−0.93 to 0.603	0.446	0.6608
	SCC [+]	−3.88	3.22	−10.58 to 2.83	1.21	0.2420
	Sheeting [+]	2.97	2.76	−2.78 to 8.72	1.1	0.2944
	Necrosis [+]	1.05	3.73	−6.73 to 8.82	0.28	0.7820

**Table A4:** Comparison of ARID1A concentrations in tumors with and without loss of heterozygosity (LOH) of chromosome 1p in an adjusted model. The ARID1A concentrations are shown, with the mean or median values in columns 2 and 3. This model for calculation includes correction for chromatin background. Means are calculated/used due to the Gaussian distribution, and Student’s *t*-test is feasible. Column 4 contains the absolute difference with indication of the standard error of the mean (SEM), as the mean is statistically regarded as a point estimate of the basic total of all tumors with and without LOH 1p. For non-Gaussian-distributed groups, medians are compared. Column 5 shows relative differences in addition to absolute value, as in the previous column. Significance levels are given in bold; (*) indicates a *p*-value < 0.05.

Fraction	Mean No LOH 1p (ng/mL)	Mean LOH 1p (ng/mL)	Difference ± SEM (ng/mL)	Relative Difference (%)	*p*-Value
Chromatin	3.0	2.35	−0.67 ± 0.31	−22.3%	**0.0345 (*)**
Nucleus	9.24	6.67	−2.56 ± 1.11	−27.7%	**0.0246 (*)**
Total	12.32	9.59	−2.74 ± 1.37	−22.2%	**0.0498 (*)**

**Table A5:** Multivariate analysis of ARID1A concentrations by several variables. Multiple linear regression is used to determine whether any of the variables ‘Sex’, ‘WHO grade’, ‘Recurrence’, ‘Localization’, or ‘LOH 1p’. As reference we chose to women, while male ‘[M]’ is regarded as influence parameter; low WHO-grade 2 is compared to higher WHO-grade 3’ ‘’; non-recurrent meningiomas are compared to recurrent ‘[R]’; single lesions were compared to multifocal ‘[Mu]’; and retained chromosome 1p is compared to ‘[LOH 1p]‘. Estimates, standard errors, 95% confidence intervals (CIs) and t-values ‘|t|’ as well as *p*-values (significance levels (*) = *p* < 0.05 and (**) = *p* < 0.01) are shown in bold for each variable and for each of the six subcellular groups (cytosol, membrane, free nucleus, chromatin, nucleus, and total). For chromatin fractions we evaluated adjusted values as described in [30].

Fraction	Variable	Estimate	Standard Error	95% CI (Asymptotic)	|t|	*p*-Value
Cytosol	Sex [M]	0.340	0.198	−0.0564 to 0.737	1.72	0.0912
	WHO grade [2]	0.269	0.223	−0.178 to 0.715	1.21	0.2333
	WHO grade [3]	−0.148	0.374	−0.898 to 0.602	0.396	0.6938
	Recurrence [R]	0.0951	0.254	−0.414 to 0.605	0.374	0.7096
	Localization [Mu]	**−0.858**	**0.306**	**−1.47 to −0.245**	**2.81**	**0.0070 (**)**
	LOH 1p [LOH]	−0.151	0.205	−0.562 to 0.259	0.740	0.4628
Membrane	Sex [M]	0.0496	0.214	−0.385 to 0.484	0.231	0.8183
	WHO grade [2]	0.184	0.250	−0.323 to 0.692	0.737	0.4660
	WHO grade [3]	−0.343	0.442	−1.24 to 0.553	0.776	0.4426
	Recurrence [R]	−0.0342	0.290	−0.622 to 0.553	0.118	0.9066
	Localization [Mu]	0.162	0.439	−0.728 to 1.05	0.369	0.7140
	LOH 1p [LOH]	0.225	0.214	−0.210 to 0.660	1.05	0.3008
Free nucleus	Sex [M]	0.345	0.929	−1.52 to 2.21	0.372	0.7117
	WHO grade [2]	−0.813	1.08	−2.98 to 1.35	0.754	0.4542
	WHO grade [3]	−0.549	1.72	−3.99 to 2.89	0.320	0.7500
	Recurrence [R]	−0.705	1.20	−3.12 to 1.71	0.586	0.5604
	Localization [Mu]	−2.17	1.51	−5.20 to 0.860	1.44	0.1567
	LOH 1p [LOH]	−1.60	0.954	−3.52 to 0.315	1.68	0.0996
Chromatin	Sex [M]	0.0378	0.310	−0.583 to 0.658	0.122	0.9032
	WHO grade [2]	−0.206	0.349	−0.905 to 0.493	0.591	0.5569
	WHO grade [3]	0.325	0.586	−0.849 to 1.50	0.555	0.5813
	Recurrence [R]	−0.451	0.398	−1.25 to 0.346	1.13	0.2616
	Localization [Mu]	−0.429	0.478	−1.39 to 0.530	0.897	0.3738
	LOH 1p [LOH]	**−0.701**	**0.320**	**−1.34 to −0.0591**	**2.19**	**0.0329 (*)**
Chromatin corrected	Sex [M]	0.304	0.341	−0.381 to 0.988	0.891	0.3771
	WHO grade [2]	−0.466	0.381	−1.23 to 0.298	1.22	0.2262
	WHO grade [3]	0.442	0.635	−0.831 to 1.72	0.697	0.4891
	Recurrence [R]	−0.741	0.442	−1.63 to 0.145	1.68	0.0995
	Localization [Mu]	−0.169	0.558	−1.29 to 0.949	0.303	0.7629
	LOH 1p [LOH]	−0.464	0.348	−1.16 to 0.234	1.33	0.1881
Nucleus	Sex [M]	0.368	1.14	−1.92 to 2.65	0.323	0.7479
	WHO grade [2]	−1.47	1.28	−4.04 to 1.11	1.14	0.2584
	WHO grade [3]	−0.409	2.16	−4.73 to 3.91	0.190	0.8504
	Recurrence [R]	−0.774	1.46	−3.71 to 2.16	0.529	0.5993
	Localization [Mu]	−2.90	1.76	−6.43 to 0.628	1.65	0.1051
	LOH 1p [LOH]	−1.89	1.18	−4.25 to 0.475	1.60	0.1150
Total	Sex [M]	0.882	1.40	−1.92 to 3.69	0.630	0.5312
	WHO grade [2]	−1.21	1.58	−4.38 to 1.95	0.771	0.4443
	WHO grade [3]	−0.910	2.65	−6.22 to 4.40	0.344	0.7325
	Recurrence [R]	−0.819	1.80	−4.43 to 2.79	0.455	0.6506
	Localization [Mu]	−4.22	2.16	−8.56 to 0.118	1.95	0.0563
	LOH 1p [LOH]	−1.77	1.45	−4.67 to 1.14	1.22	0.2275
